# Whole-Genome Sequence of Brevibacillus borstelensis SDM, Isolated from a Sorghum-Adapted Microbial Community

**DOI:** 10.1128/MRA.01046-20

**Published:** 2020-11-25

**Authors:** Martina Aulitto, Lauren M. Tom, Javier A. Ceja-Navarro, Blake A. Simmons, Steven W. Singer

**Affiliations:** a Joint BioEnergy Institute, Emeryville, California, USA; b Biological Systems and Engineering Division, Lawrence Berkeley National Laboratory, Berkeley, California, USA; Georgia Institute of Technology

## Abstract

The isolation of novel microbes from environmental samples continues to be a key strategy for the discovery of new metabolic capacities for the degradation and transformation of lignocellulose. We report the draft genome sequence of a new strain of Brevibacillus borstelensis isolated from a sorghum-adapted microbial community derived from a compost sample.

## ANNOUNCEMENT

Soil contains an untapped diversity of microbial communities with equally diverse metabolic capacities (1). Researchers around the world continue developing strategies for isolating microorganisms that can efficiently degrade lignocellulosic biomass, and most rely on the isolation of these microorganisms from environmental samples (2). In the present study, a new strain of Brevibacillus borstelensis was isolated from a compost-derived microbial community enriched on untreated sorghum biomass. To date, several *B. borstelensis* organisms have been sequenced, but only a few have available whole-genome sequences (BioProject numbers PRJDB5988, PRJNA191598, PRJNA229942, PRJNA233554, PRJNA348753, and PRJNA498706) (3, 4). *B. borstelensis* is a Gram-positive, aerobic, thermophilic, endospore-forming bacterium of the family *Paenibacillaceae*. Other studies have shown the importance of *B. borstelensis* in different environmental applications, such as the capability to degrade polyethylene by 30% (5) and to enhance the transformation of food waste into biofertilizer (6).

*B. borstelensis* SDM was isolated by spreading an enrichment culture of green waste compost obtained from the city of Berkeley, California, incubated with sorghum in M9TE (7) on Luria Bertani (LB) solid medium and incubating it overnight at 50°C. After several isolation steps, six colonies were randomly picked and identified through 16S rRNA-encoding gene sequencing as Brevibacillus borstelensis (99% identical). *B. borstelensis* was cultivated overnight in LB medium (at 50°C and 200 rpm), and genomic DNA was extracted using a DNeasy PowerSoil kit (Qiagen). The library was prepared using a KAPA HyperPrep kit for DNA (KK8504) and then sequenced with a NovaSeq S4 platform (Illumina). The raw sequences were uploaded to KBase (https://narrative.kbase.us/narrative/ws.39490.obj) (8), and default parameters were used for all software unless otherwise noted. The read quality was assessed using FastQC v 0.11.5 (9), and a quality score above Q30 for 97.9% of the bases was reported. A total of 611,322,296 reads (average length, 151 bp) were assembled using (3) different assemblers (SPAdes v 3.13.0 [10], IDBA-UD v 1.1.3 [11], and MEGAHIT v 1.2.9 [12]) and compared using QUAST v 4.4 (13). The best assembly was obtained using MEGAHIT and comprised 128 contigs, all of them with ≥1,000 bp, with a total length of 5,246,051 bp, an *N*_50_ value of 82,015 bp, and an average GC content of 51.62%. The genome coverage was 116.5×. Furthermore, the assembly was evaluated for completeness (99.73%) and contamination (2.35%) using CheckM v 1.0.18 (14).

The genome annotation of *B. borstelensis* SDM was performed using the NCBI Prokaryotic Genome Annotation Pipeline (PGAP), where a total of 4,853 coding sequences (CDS) were identified (15). Moreover, for the annotation of carbohydrate-active enzymes (CAZYmes), all proteins were mapped against the dbCAN database v 8 (16) using hmmscan (HMMER 3.1b2) with an E value cutoff of 1e-5. This analysis revealed the existence of 266 CAZYmes, suggesting that *B. borstelensis* SDM has the potential to degrade and transform lignocellulosic biomass.

### Data availability.

This whole-genome shotgun project has been deposited at DDBJ/ENA/GenBank under the accession number JABTBQ000000000, and the raw reads have been deposited in the Sequence Read Archive under the accession number PRJNA633907.
